# A placenta-on-a-chip model to determine the regulation of FKBPL and galectin-3 in preeclampsia

**DOI:** 10.1007/s00018-022-04648-w

**Published:** 2023-01-18

**Authors:** Sahar Masoumeh Ghorbanpour, Claire Richards, Dillan Pienaar, Kimberly Sesperez, Hamidreza Aboulkheyr Es., Valentina N. Nikolic, Natasa Karadzov Orlic, Zeljko Mikovic, Milan Stefanovic, Zoran Cakic, Abdelrahim Alqudah, Louise Cole, Catherine Gorrie, Kristine McGrath, Mary M. Kavurma, Majid Ebrahimi Warkiani, Lana McClements

**Affiliations:** 1 School of Life Sciences, Faculty of Science, University of Technology Sydney, Sydney, NSW Australia; 2 Institute for Biomedical Materials and Devices, Faculty of Science, University of Technology Sydney, Sydney, NSW Australia; 3 School of Biomedical Engineering, Faculty of Engineering and Information Technology, University of Technology Sydney, Sydney, NSW Australia; 4 Department of Pharmacology and Toxicology, Faculty of Medicine, University of Nis, Niš, Serbia; 5 Department of Internal Medicine-Gynaecology, Faculty of Medicine, University of Nis, Niš, Serbia; 6 Department of Gynaecology and Obstetrics, Narodni Front, Belgrade, Serbia; 7 Faculty of Medicine, University of Belgrade, Belgrade, Serbia; 8 Department of Gynaecology and Obstetrics, Clinical Centre Nis, Niš, Serbia; 9 Department of Gynaecology and Obstetrics, General Hospital of Leskovac, Leskovac, Serbia; 10 Department of Clinical Pharmacy and Pharmacy Practice, Faculty of Pharmaceutical Sciences, The Hashemite University, Zarqa, Jordan; 11 Australian Institute of Microbiology and Infection, Faculty of Science, University of Technology Sydney, Sydney, NSW Australia; 12 Heart Research Institute, Sydney, NSW Australia; 13 Heart Research Institute,The University of Sydney, Sydney, NSW Australia

**Keywords:** Microfluidics, Placental development, Preeclampsia, Vascular remodeling, FKBPL, Galectin-3

## Abstract

Preeclampsia is a pregnancy-specific cardiovascular disorder, involving significant maternal endothelial dysfunction. Although inappropriate placentation due to aberrant angiogenesis, inflammation and shallow trophoblast invasion are the root causes of preeclampsia, pathogenic mechanisms are poorly understood, particularly in early pregnancy. Here, we first confirm the abnormal expression of important vascular and inflammatory proteins, FK506-binding protein-like (FKBPL) and galectin-3 (Gal-3), in human plasma and placental tissues from women with preeclampsia and normotensive controls. We then employ a three-dimensional microfluidic placental model incorporating human umbilical vein endothelial cells (HUVECs) and a first trimester trophoblast cell line (ACH-3P) to investigate FKBPL and Gal-3 signaling in inflammatory conditions. In human samples, both circulating (*n* = 17 controls; *n* = 30 preeclampsia) and placental (*n* ≥ 6) FKBPL and Gal-3 levels were increased in preeclampsia compared to controls (plasma: FKBPL, *p* < 0.0001; Gal-3, *p* < 0.01; placenta: FKBPL, *p* < 0.05; Gal-3, *p* < 0.01), indicative of vascular dysfunction in preeclampsia. In our placenta-on-a-chip model, we show that endothelial cells are critical for trophoblast-mediated migration and that trophoblasts effectively remodel endothelial vascular networks. Inflammatory cytokine tumour necrosis factor-α (10 ng/mL) modulates both FKBPL and Gal-3 signaling in conjunction with trophoblast migration and impairs vascular network formation (*p* < 0.005). Our placenta-on-a-chip recapitulates aspects of inappropriate placental development and vascular dysfunction in preeclampsia.

**Supplementary Information:**

The online version contains supplementary material available at 10.1007/s00018-022-04648-w.

## Introduction

Preeclampsia is characterized by the new onset of high blood pressure in combination with proteinuria or organ dysfunction and can be categorized into an early-onset (diagnosed < 34-week gestation), late-onset (diagnosed ≥ 34 weeks) or delayed postpartum phenotype [1–3]. In severe cases, preeclampsia can be life threatening during pregnancy and also leads to increased risk of both the mother and offspring developing cardiovascular and metabolic disorders later in life [4–7]. Currently, there are limited monitoring options for women at risk of preeclampsia and the only definitive treatment is the delivery of the placenta and the baby, which is often preterm and associated with many complications. Though much of the etiology of preeclampsia is unknown, it likely has origins in inappropriate placentation and vascular dysfunction leading to systemic oxidative stress, inflammation, endothelial dysfunction and an anti-angiogenic environment [8].

During normal placental development, trophoblast cells invade the decidualized endometrial lining of the uterus to anchor the placenta and establish connection to the maternal circulation. An invasive subtype of trophoblasts, extravillous trophoblasts (EVTs), invade the spiral uterine arteries (SUAs) of the decidua and inner third of the myometrium in a tightly regulated fashion [9]. These vessels are subsequently remodeled, replacing the endothelial and muscle layers to reduce vessel resistance, resulting in unrestricted blood flow, which facilitates the growth of the fetus during pregnancy [10]. However, impaired SUA remodeling has been observed in women with preeclampsia and is believed to play a key role in its pathogenesis, triggering a cascade of events following placental malperfusion [11]. For example, it has been shown that preeclampsia is associated with upregulation of inflammatory factors including tumor necrosis factor alpha (TNF-α) and interleukin 6 (IL-6); cell adhesion molecules, including soluble vascular cell adhesion molecule 1 (sVCAM-1) and intercellular adhesion molecule 1 (sICAM-1); cellular fibronectin and anti-angiogenic proteins, including soluble fms-like tyrosine kinase-1 (sFlt-1), amongst others [8, 12–21]. Two emerging pathways, anti-angiogenic FK506-binding protein-like (FKBPL) and pro-inflammatory galectin-3 (Gal-3), have been implicated in preeclampsia and associated cardiovascular complications [22, 23].

FKBPL belongs to the immunophilin protein group and has been shown to have roles in the regulation of glucocorticoid, androgen and estrogen receptor signaling, stem cell differentiation, inflammation and inhibition of angiogenesis; the latter three mediated via the CD44 cell surface receptor [24–32]. Furthermore, recent data have demonstrated that FKBPL could be used as a diagnostic and/or treatment target for cardiovascular diseases (CVDs), placental health and preeclampsia [22, 33–35]. We recently showed that plasma and placental FKBPL expression levels were significantly higher in a cohort of pregnant women with preeclampsia compared to normotensive pregnancies. We demonstrated that FKBPL expression analyzed against its target, CD44, as a CD44/FKBPL ratio, could be used as a potential predictive and diagnostic tool for preeclampsia, although its role in the pathogenesis of preeclampsia requires further elucidation.

In addition, Gal-3 is a cytokine-like, immunoregulatory protein that is involved in various pathologies associated with inflammation including heart remodeling/disease and has been shown to have potential as a biomarker of cardiovascular disease risk in women following preeclampsia [23, 36, 37]. The distribution of Gal-3 in normal and malignant trophoblasts [38–40], as well as its important roles in tissue remodeling [36, 37], tumor cell adhesion [41], cancer immune evasion [42], epithelial wound healing, cell migration [43–45], proliferation [46], and angiogenesis[47] have previously been demonstrated. While some reports have identified an upregulation of Gal-3 in plasma and placental samples of women with preeclampsia vs normotensive pregnancies [48, 49], its mechanisms in the pathogenesis of this disease and future cardiovascular disease risk require further investigation [50].

Given the challenges with investigating the early placentation processes in humans due to increased miscarriage risk associated with chorionic villus sampling, mechanisms of placental development and growth are still poorly understood. Traditional two-dimensional (2D) in vitro assays and in vivo models are limited due to their simplicity and inter-species differences, respectively [51–55]. Three-dimensional (3D) cell culture models more closely recapitulate human tissue to study cell–cell and cell–matrix interactions and specifically, microfluidic devices and organ-on-a-chip platforms allow the dynamic in vitro representation of various physiological processes. 3D models can facilitate better understanding of cellular and molecular mechanisms of aberrant placentation and provide a platform for therapeutic screening for pregnancy-related disorders. Some microfluidic models have already been developed to represent placental cell interactions, as recently reviewed by Richardson et al*.* and Young et al. [56, 57], with some of these perhaps lacking relevant cell types.

In this study, we aimed to design a 3D microfluidic placental model incorporating human umbilical vein endothelial cells (HUVECs) and first trimester trophoblast cell line, ACH-3P, which was developed by the fusion of primary first trimester trophoblasts with an established choriocarcinoma cell line. This line has been shown to differentiate into at least two distinct trophoblast subpopulations; from human leukocyte antigen-G (HLA-G) negative villous cytotrophoblasts into HLA-G positive EVTs. ACH-3P cultures can also form an autocrine and paracrine regulatory loop [58]. This placenta-on-a-chip 3D microfluidic platform was used to investigate (i) trophoblast–endothelial cell interactions during placental development, (ii) endothelial cell network formation and (iii) FKBPL and Gal-3 mechanisms during these processes, particularly within inflammatory conditions, which are characteristic of preeclampsia.

## Materials and methods

### Human sample collection

Human plasma and placental samples were collected as part of a multicenter study including three hospitals in Serbia. Blood samples were collected from 47 participants from maternal age-matched, normotensive healthy control pregnancies without any pre-existing conditions or pregnancy complications (*n* = 17) and pregnant women with preeclampsia (*n* = 30), prior to labor. Women with multiple pregnancies were excluded from the study. Blood samples were collected in ethylenediaminetetraacetic acid (EDTA) tubes and centrifuged at 3000 g for 10 min at 4 °C to isolate plasma. The samples were stored at − 80 °C. The placentae were dissected following delivery, and two full thickness blocks (2 × 2 cm) were collected and transferred to a − 80 °C freezer. Placental samples were chosen at random (*n* = 6 controls and *n* = 11 from women with preeclampsia) for determining protein expression of Gal-3 and FKBPL. Preeclampsia was defined according to the American College of Obstetricians and Gynecologists (ACOG) 2019 guidelines [59]. Clinical characteristics of maternal age, gestational age, maternal body mass index (BMI), systolic (sBP), diastolic (dBP) and mean arterial blood pressure (MABP) and gravidity are presented in Supplementary Information (Tables 1, 2) according to sample group.

### Protein extraction from placental samples

Placental tissue (100 mg) was homogenized with beads using 250 µl of RIPA lysis buffer (50 mM Tris–HCL, 150 mM NaCl, 0.1% Triton, 0.5% Sodium deoxycholate, 0.1% SDS, pH 8) containing 1% Halt Protease Inhibitor Cocktail (Thermo Fisher Scientific, USA) and incubated on ice for 30 min before samples were centrifuged at 14,000 rpm for 10 min at 4 °C. The supernatant was collected and stored at − 80 °C for downstream analysis.

### Cell culture

HUVECs (Promocell, Germany) were maintained in microvascular endothelial cell growth media (EGMTM-2MV, Lonza, Switzerland). The ACH-3P trophoblast cells were kindly donated by Professor Gernot Desoye (Graz Medical University, Austria) and were maintained in Ham’s F12 nutrient mix supplemented with 10% fetal bovine serum (FBS) and 1% penicillin–streptomycin (Thermo Fisher Scientific, UK). Both cell types were incubated in a 37 °C humidified atmosphere with 5% CO_2_. Flasks approaching 90% confluency were passaged using 1% Trypsin (Thermo Fisher Scientific, USA) for HUVECs and StemPro Accutase (Thermo Fisher Scientific, UK) for ACH-3Ps. Every five passages, ACH-3P cells were treated with a selection medium containing azaserine (5.7 μM) and hypoxanthine (100 μM) to prevent the overgrowth of choriocarcinoma.

### 2D in vitro ACH-3P and HUVEC TNF-α treatment

ACH-3Ps or HUVECs were seeded at 200,000 cells/well in 12-well plates and incubated at 37 °C and 5% CO_2_. When they had reached 90% confluency, cells were starved for 6 h in their respective medium containing 1% FBS. Cells were treated with 10 ng/mL TNF-α (as previously described [58, 60–62]; Sigma-Aldrich, USA, cat. #T0157) for 24 or 72 h with untreated cells used as a control. The cells for the control at both 24- and 72-h timepoints were seeded and grown for the same period of time. Intracellular protein was extracted using 100 μl/well of RIPA lysis buffer and processed as described for tissue protein extraction above.

### 3D-Microfluidic model of placenta

Microfluidic tissue culture devices were purchased from AIM Biotech (Singapore). These plastic devices are composed of a cyclic olefin polymer (COP) chip body laminated with a gas-permeable laminate. The devices consist of three microfluidic chambers each with two parallel side media channels (width 0.5 mm) and a central region called the gel channel (width 1.3 mm) with a height of 0.25 mm. Interstitial flow within the device is generated by adding different media volumes in opposing media channels, creating a pressure gradient. Three experimental settings were employed: (i) ACH-3Ps monoculture, (ii) HUVECs monoculture and (iii) ACH-3P and HUVEC co-culture. An extracellular matrix (ECM) solution containing 2.5 mg/ml collagen type I (Thermo Fisher Scientific, UK, cat. #A1048301), 10× PBS, H_2_O and NaOH (0.5 N) at pH 7.4, was prepared and kept on ice to avoid polymerization. In setting (i), the ECM was injected into the dedicated gel region of the device according to the manufacturer’s protocols [63] and the device was incubated at 37 °C and 5% CO_2_ for 40 min to allow gel polymerization via thermal cross‐linking. ACH-3Ps (2 × 10^6^/mL) were added to one of the side media channels and incubated at 37 °C and 5% CO_2_ overnight. In setting (ii), the ECM solution was mixed with 8 × 10^6^/mL HUVECs on ice and added to the central gel channel prior to polymerization. In setting (iii), the procedures in settings (i) and (ii) were performed in combination. In each setting, the media side channels were filled with 120 µL EGM™-2MV from the top media inlet and 60 µL from the bottom inlet immediately after gel polymerization and the chips were incubated at 37 °C and 5% CO_2_. The media was changed every 24 h. Cells were also treated with 10 ng/mL TNF-α for 24 or 72 h, with untreated cells as a treatment control.

### Immunofluorescence staining

Cell culture medium was removed from the microfluidic devices prior to washing with PBS. Cells were fixed with 4% paraformaldehyde (PFA; Sigma‐Aldrich, USA) for 15 min at 37 °C followed by permeabilization with 0.1% Triton‐X (Sigma‐Aldrich, USA) for 10 min at room temperature. To reduce non-specific binding, cells were blocked with blocking buffer containing 5% bovine serum albumin (Sigma-Aldrich, USA) and 3% normal goat serum (Invitrogen, USA) for 4 h at room temperature. The cells were then probed with a combination of either Gal-3 (1:200; R&D Systems, USA, cat. #842759), FKBPL (1:200; Proteintech, USA, cat. #100601AP), CD31 (1:200; Abcam, UK, cat. #ab24590), EpCAM (1:200; Genesearch, AU, cat. #D1B3), HLA-G (1:200; Bio-Rad, USA, cat. #MCA2043) and Cytokeratin-7 (1:200; Abcam, UK, cat. #ab181598) primary antibodies and incubated at 4 °C overnight. Following washing, goat anti-rabbit IgG H&L (Alexa Fluor^®^ 488, Abcam, cat. #150077) and goat anti-mouse IgG H&L (Alexa Fluor^®^ 594, Abcam, cat. #150116) secondary antibodies were added for 2 h at room temperature. Finally, the nuclei were stained with DAPI (10 µg/mL, Invitrogen, USA, cat. #D1306) for 1 h at room temperature, prior to washing five times with PBS and stored at 4 °C.

### Widefield and laser scanning confocal microscopy

Fluorescence images were obtained using Leica Stellaris confocal and Nikon TiE2 widefield fluorescence microscopes. Confocal images were acquired using a 20× objective with NA 1.45 and Nyquist sampling. Z stacks (0.2 µm optical slices) were acquired using a 0.5 AU pinhole. Widefield images were acquired with a 20× objective with NA 0.75 and long working distance (2300 µm). Images were either deconvolved with NIS-Elements (version 5.3) using Richardson–Lucy method or clarified using NIS-Elements Clarify.ai [64]. The intensity of fluorescent signal, an indicator of protein expression, was analyzed using ImageJ software (NIH, USA, version 2.1.0) on maximum intensity projection images. Five images from each device were analyzed and averaged and the fluorescent intensity was normalized to the nuclear count. Network branching of endothelial vascular networks was analyzed using the Angiogenesis Analyzer macro on ImageJ [65].

### Western blotting

Human placental and cell lysate protein samples were quantified using a bicinchoninic acid (BCA) assay (Thermo Scientific Pierce ™ BCA Protein Assay Kit, #23225). Samples (20 µg) were reduced with 4X Laemmli sample buffer (Bio-Rad, USA) and subjected to Western Blotting. Membranes were probed with mouse anti-FKBPL (1:1000; Proteintech, USA, cat. #663891Ig), mouse anti-human galectin-3 (1:500; R&D Systems, USA, cat. 842759), or rabbit anti-Glyceraldehyde 3-phosphate dehydrogenase (GAPDH; 1:6000; Abcam, UK, cat. #ab37168) primary antibodies in 5% skim milk overnight at 4 °C. Membranes were washed and incubated with their corresponding secondary antibody, anti-mouse IgG (1:1,000; GE Healthcare, UK, cat. # NXA931) or anti-rabbit (Abcam, UK, cat. #ab6721) antibody, and imaged by chemiluminescence using a ChemiDoc MP imaging system (Bio-Rad, USA). Band intensity was measured using ImageJ and normalized to the housekeeping protein, GAPDH.

### FKBPL ELISA

Plasma from women with preeclampsia was analyzed for FKBPL concentration by sandwich ELISA and compared to that of normotensive controls. An FKBPL ELISA kit from Cloud-Clone Corp (#SEL523Hu, China) was used according to the manufacturer’s instructions. Optical density was measured using a Spark 10 M plate reader (Tecan, Switzerland) at 450 nm. The four-parameter logistic (4PL) curve regression model was used to determine concentration values of each sample from the sigmoidal standard curve.

### Galectin-3 ELISA

The Gal-3 protein concentration of placental samples was quantified using a galectin-3 DuoSet enzyme-linked immunosorbent assay (ELISA) kit (R&D Systems, USA, cat. #DY1154) according to the manufacturer’s instructions. The absorbance was measured at 450 nm and 540 nm (reference wavelength) using a Spark 10 M plate reader (Tecan, Switzerland). The four-parameter logistic (4PL) curve regression model was used to determine concentration values of each sample from the sigmoidal standard curve.

### Statistical analysis

The results of human sample quantifications were presented as mean ± SD, whereas the results of quantitative in vitro experiments were presented as mean ± SEM. Normality testing was performed using a Shapiro–Wilks test followed by two-tailed unpaired *t* test, one-way ANOVA or two-way ANOVA with post hoc multiple comparison tests. For non-normally distributed data, Mann–Whitney or Kruskal–Wallis were used. Statistical analysis was performed using GraphPad Prism (version 8.4.3 software, USA) and *p* value < 0.05 was considered statistically significant. An unpaired t test was used to determine differences between gestational age, maternal age and BMI. Where there were statistically significant differences between the groups (*p* < 0.05), SPSS software (IBM 1.0.0.146, USA) was used to perform correlations between preeclampsia and FKBPL or Gal-3 plasma concentration or placental expression using Pearson’s correlation and partial correlation controlling for these factors.

## Results

### Circulating and placental FKBPL and Gal-3 are increased in preeclampsia

**Fig. 1:**
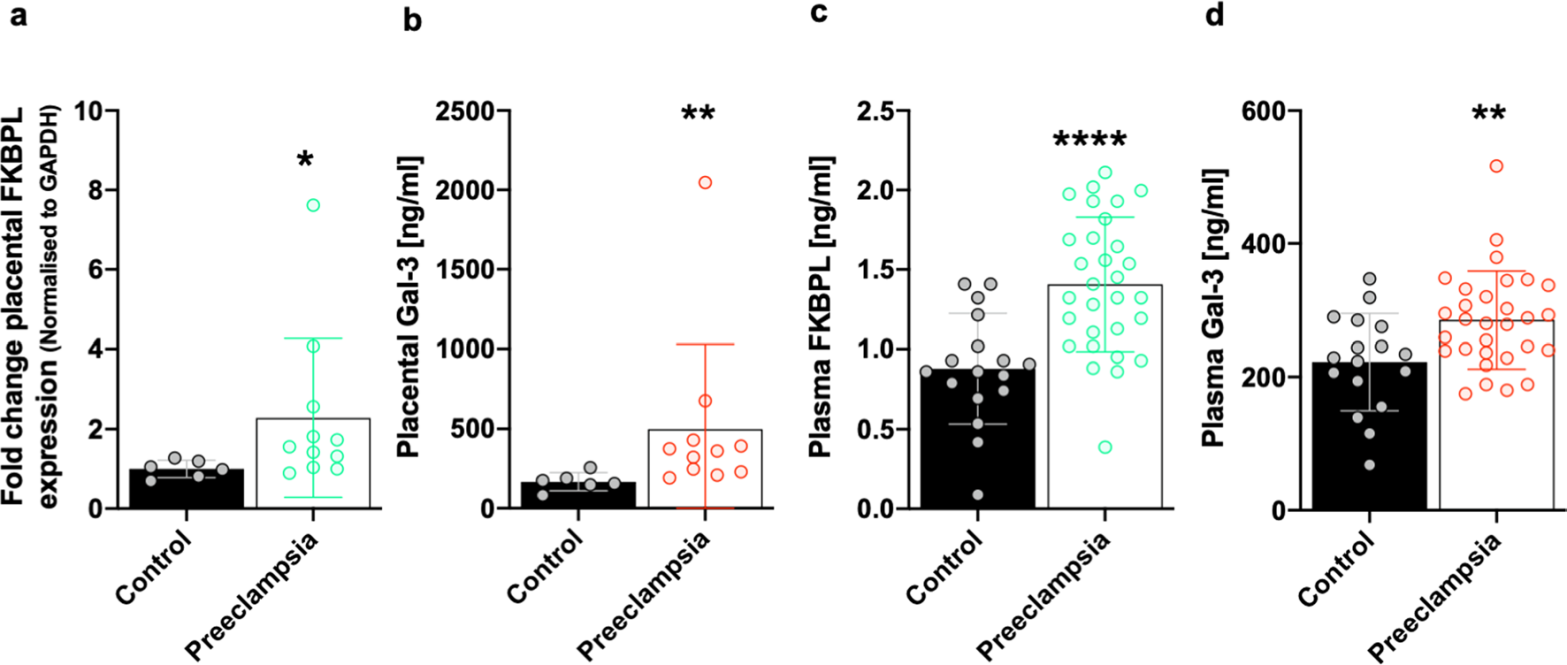
FKBPL and Gal-3 are increased in the placentae and plasma of women with preeclampsia. Protein lysates were generated from placental tissue collected from women with preeclampsia or normotensive controls. **a** FKBPL expression was determined by Western Blotting and normalized to GAPDH, the loading control. **b** Gal-3 levels from placental lysates were evaluated by enzyme-linked immunosorbent assay (ELISA). Data were plotted as mean ± SD; *n* ≥ 6. **c**, **d** Plasma FKBPL and Gal-3 levels from women with preeclampsia vs normotensive controls were assessed by ELISA. Data plotted as mean ± SD; *n* ≥ 17; unpaired student’s *t* test, *p < 0.05, **p < 0.01, ***p < 0.001, ****p < 0.0001

Given FKBPL is an intracellular protein that is lowly secreted, predominantly by endothelial and fibroblast cells [28], it was deemed appropriate to analyse FKBPL in placental tissue by Western Blotting and secreted FKBPL in plasma by ELISA. Gal-3 expression in placental tissue and plasma was analysed using ELISA. With respect to variations between case (preeclampsia) and control groups, no differences in BMI were observed; however, gestational age was significantly lower and maternal age significantly higher in the preeclampsia group (Supplementary Table 1). Placental FKBPL protein expression was over two-fold higher from women with preeclampsia compared to normotensive controls (control 1.00 ± 0.22 vs preeclampsia 2.28 ± 1.99, fold change, *p* = 0.02; Fig. 1a and Supplementary Fig. 1). Although no correlation was observed between placental FKBPL protein expression and preeclampsia (*r* = 0.370, *p* = 0.144, Table 1), this became statistically significant after adjusting for confounders including gestational and maternal age (*r* = 0.519, *p* = 0.047, Table 1). Similarly, placental Gal-3 protein expression was increased in the preeclampsia group (control 167.4 ± 56.7 vs preeclampsia 498.2 ± 531.5, pg/mL, *p* = 0.004; Fig. 1b); however, no correlation between placental Gal-3 expression and preeclampsia was observed (*r* = 0.361, *p* = 0.155, Table 1), even when adjusted for gestational and maternal age (*r* = 0.356, *p* = 0.193; Table 1).

**Table 1:** Adjusted correlations between FKBPL/Gal-3 and preeclampsia for differences in gestational age and maternal age between normotensive and preeclamptic placentae

Placental samples	FKBPL		Gal-3	
	Pearson Correlation	Correlation controlled by GA and MA	Pearson Correlation	Correlation controlled by GA and MA
PE	*r* = 0.370	*r* = 0.519	*r* = 0.361	*r* = 0.356
	*p* = 0.144	***p* = 0.047**	*p* = 0.155	*p* = 0.193

Bold indicates statistical significance (*p* < 0.05)

*FKBPL* FK506-binding protein-like, *Gal-3* galectin-3, *PE* preeclampsia, *GA* gestational age, *MA* maternal age

While there were no differences in maternal age between the normotensive and preeclampsia plasma samples, gestational age was significantly lower and body mass index (BMI) significantly higher in the preeclampsia group (Supplementary Table 2). Plasma samples analysed by ELISA demonstrated a significant increase in FKBPL concentration in women with preeclampsia compared to normotensive pregnancies (control 0.88 ± 0.35 vs preeclampsia 1.41 ± 0.42, ng/mL, *p* < 0.0001; Fig. 1c). Furthermore, there was a significant positive correlation between plasma FKBPL and preeclampsia (*r* = 0.578, *p* < 0.001), even when adjusted for differences in gestational age and BMI (*r* = 0.559, *p* < 0.001; Table 2). Similarly, plasma Gal-3 concentration from women with preeclampsia compared to controls was also increased (control 222.2 ± 72.91 vs preeclampsia 288.8 ± 71.98, pg/mL, *p* = 0.004; Fig. 1d). Aligned to this, plasma Gal-3 concentration was positively correlated with preeclampsia (*r* = 0.389, *p* = 0.007), although the statistical significance was lost when adjusted for differences in gestational age and BMI as confounding factors (*r* = 0.281, *p* = 0.064; Table 2).

**Table 2:** Adjusted correlations between FKBPL/Gal-3 and preeclampsia for differences in gestational age and BMI between plasma samples from pregnant women with preeclampsia or normotensive pregnancies

Plasma samples	FKBPL		Gal-3	
	Pearson Correlation	Correlation controlled by GA and BMI	Pearson Correlation	Correlation controlled by GA and BMI
PE	*r* = 0.578	*r* = 0.559	*r* = 0.389	*r* = 0.281
	***p* < 0.001**	***p* < 0.001**	***p* = 0.007**	*p* = 0.064

Bold indicates statistical significance (*p* < 0.05)

*FKBPL* FK506-binding protein-like, *Gal-3* galectin-3, *PE* preeclampsia, *GA* gestational age, *BMI* body mass index

### Inflammation regulates FKBPL and Gal-3 expression in trophoblast and endothelial cell 2D monocultures

**Fig. 2:**
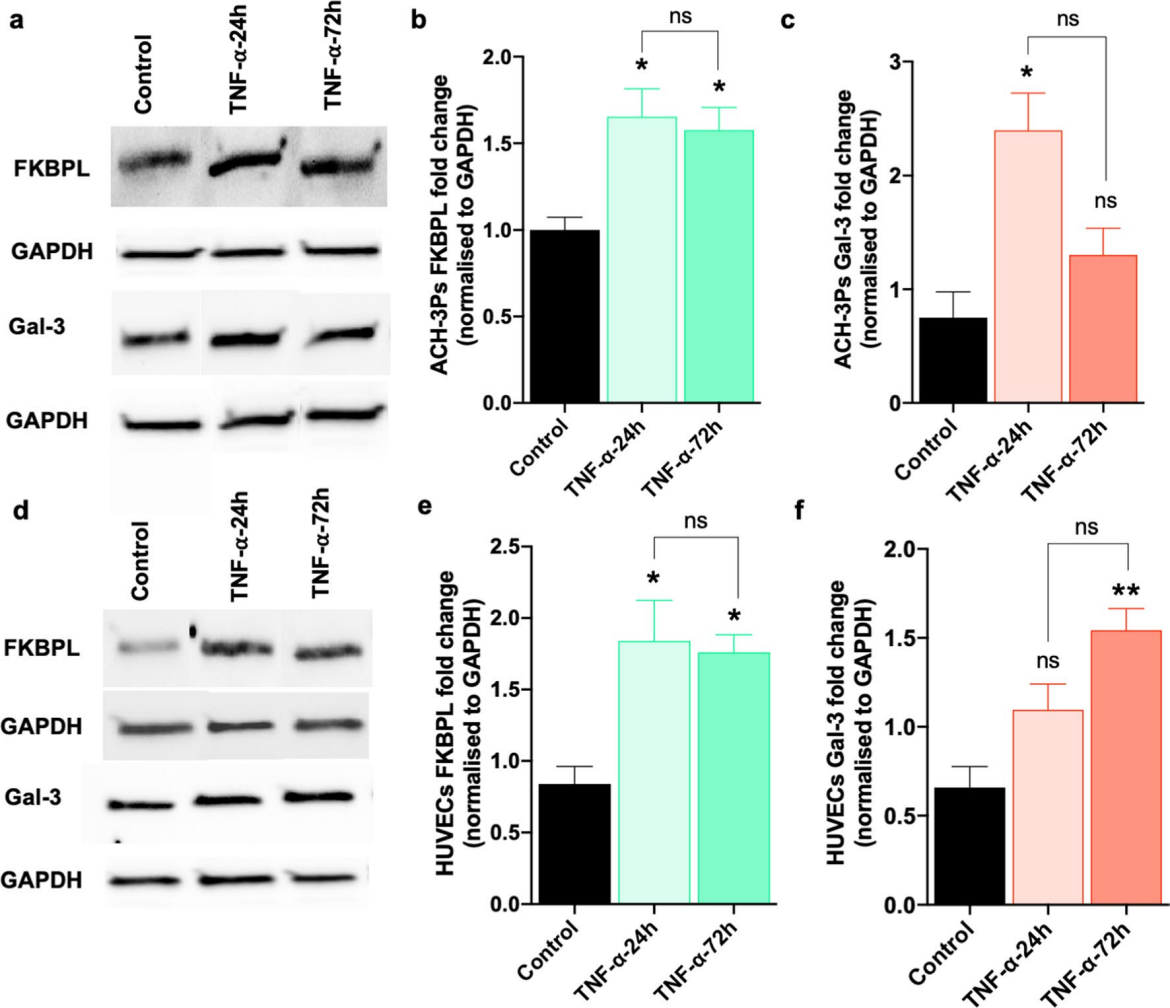
TNF-α treatment of trophoblasts and endothelial cells in 2D monocultures alters FKBPL and Gal-3 protein expression. **a**–**c** Western Blotting results of ACH-3Ps protein lysate expression of FKBPL and Gal-3. ACH-3Ps exposed to tumor necrosis factor alpha (TNF-α, 10 ng/mL) for 24 or 72 h. Control, untreated. GAPDH, loading control. **d**–**f** Western Blotting of HUVECs protein lysate showing expression of FKBPL and Gal-3. HUVECs exposed to TNF-α (10 ng/mL) for 24 or 72 h. Control, untreated GAPDH, loading control. Data passed Shapiro–Wilk normality test and were analyzed by one-way analysis of variance (ANOVA) with Tukey post-hoc test; *n* = 3; *p < 0.05, ***p < 0.001

To investigate the regulation of FKBPL and Gal-3 under inflammatory conditions, trophoblasts or endothelial cells were treated with TNF-α (10 ng/mL), an inflammatory stimulus elevated in preeclampsia [14], for 24 and 72 h. FKBPL protein expression was significantly increased ~ 1.5-fold, in ACH-3Ps at both timepoints (control 1.00 ± 0.074 vs TNF-α-24 h 1.65 ± 0.16 vs TNF-α-72 h 1.58 ± 0.13, fold change, *p* = 0.021; Fig. 2a, b and Supplementary Fig. 2). TNF-α exposure also stimulated Gal-3 protein expression by ~ 2.5-fold in ACH-3Ps exposed to 24-h TNF-α; however, this increase was non-significant by 72 h (control 0.751 ± 0.23 vs TNF-α-24 h 2.40 ± 0.33 vs TNF-α-72 h 1.3 ± 0.23, fold change, *p* = 0.012; Fig. 2a, c and Supplementary Fig. 2).

Similarly, FKBPL protein expression was increased in HUVECs ~ two-fold following 24 h of TNF-α treatment and was maintained at 72 h (control 0.84 ± 0.12 vs TNF-α-24 h 1.84 ± 0.28 vs TNF-α-72 h 1.76 ± 0.12, fold change, *p* = 0.018; Fig. 2d, e and Supplementary Fig. 3). When we examined Gal-3 protein expression in HUVECs, there was a trend towards an increase in Gal-3 following 24 h exposure to TNF-α, though this did not become significant until it increased by ~ two-fold at 72 h (control 0.65 ± 0.11 vs TNF-α-24 h 1.09 ± 0.14 vs TNF-α-72 h 1.54 ± 0.12, fold change, *p* = 0.008; Fig. 2d, f and Supplementary Fig. 3).

### Trophoblast migration is stimulated by the presence of endothelial cells or inflammatory conditions

**Fig. 3:**
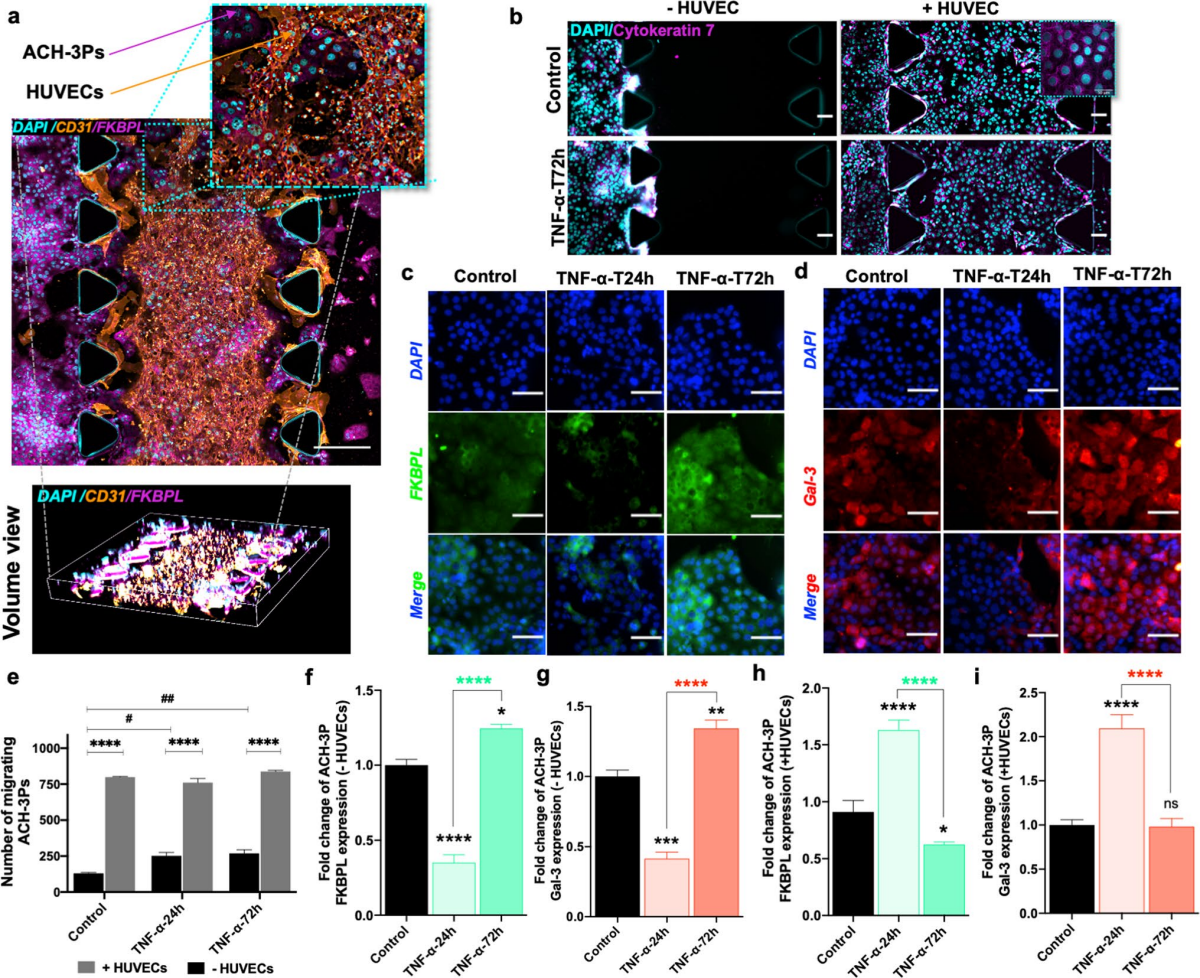
Endothelial cell presence and TNF-α modify trophoblast migration, FKBPL and Gal-3 expression in a microfluidic chip. In the co-culture set of chips, HUVECs were embedded within the center matrix channel and ACH-3Ps were added to the side channel. **a** Representative immunofluorescence (IF) images of ACH-3Ps and HUVECs with high expression for FKBPL and CD31, respectively. Nuclei of cells were visualized using DAPI. **b** Representative IF images of ACH-3Ps invasion across the device (left to right) in the absence or presence of HUVECs and in normal or inflammatory conditions. Cells were IF stained for cytokeratin 7, a marker of trophoblasts, and DAPI. **e** The number of migrating trophoblast cells from the left side channel were analyzed using ImageJ. **c**, **d** ACH-3Ps monoculture chips were also fixed and IF stained for FKBPL, Galectin-3 and DAPI. Chips were treated with TNF-α (10 ng/mL) for 24 or 72 h, with untreated cells as a control. The fold change of **f** FKBPL expression in ACH-3Ps without HUVECs and **h** with HUVECs. The fold change of **g** Gal-3 expression in ACH-3Ps without HUVECs and **i** with HUVECs Gal-3. Scalebars represent 100 µm. Data plotted as mean fold change ± SEM, ordinary one-way ANOVA or two-way ANOVA with Tukey post hoc test, *n* = 3, *p < 0.05, **p < 0.01, ***p < 0.001, ****p < 0.0001

We next used our placenta-on-a-chip model to assess the invasive and migratory ability, as well as regulation of FKBPL and Gal-3, under inflammatory conditions (a hallmark feature of preeclampsia), of trophoblasts in the absence and presence of endothelial cells (Fig. 3a). The presence of both villous and EVT populations was confirmed by labelling with EpCAM and HLA-G antibodies, respectively (Supplementary Fig. 4a, b). In the trophoblast monoculture microfluidic setting, ACH-3Ps were limited in their invasion through the collagen matrix without HUVECs presence and in the absence of TNF-α (Fig. 3b, e). TNF-α exposure stimulated a significant increase in ACH-3Ps migration at both timepoints (control 130.3 ± 11.23 vs TNF-α-24 h 252 ± 41.67 vs TNF-α-72 h 269.3 ± 43.6, *p* = 0.0039; Fig. 3b, e). FKBPL protein expression was significantly reduced with TNF-α treatment at 24 h before it was restored and increased by 72 h (control 1.00 ± 0.04 vs TNF-α-24 h 0.35 ± 0.05 vs TNF-α-72 h 1.25 ± 0.03, fold change, *p* < 0.0001; Fig. 3c, f). A similar effect was observed for Gal-3 expression in the absence of endothelial cells (control 1.00 ± 0.05 vs TNF-α-24 h 0.41 ± 0.05 vs TNF-α-72 h 1.34 ± 0.06, fold change, *p* < 0.0001; Fig. 3d, g).

In the trophoblast and endothelial co-culture microfluidic setting, ACH-3Ps actively traversed the collagen matrix across the chip, and there were no differences in ACH-3Ps’ migration exposed to inflammatory conditions (Fig. 3a, b, e). Interestingly, there was a significant increase in the number of migrating trophoblasts in the presence of endothelial cells, as determined by cytokeratin 7 staining (without HUVECs vs with HUVECs; Control: 130.3 ± 11.23 vs 799 ± 8.71, TNF-α-24 h: 252 ± 41.67 vs 761 ± 18.66, TNF-α-72 h: 269.3 ± 43.6 vs 838 ± 7.93, *p* < 0.0001; Fig. 3b, e). The presence of endothelial cells increased the FKBPL expression of trophoblasts following 24 h of TNF-α treatment, which was reduced by 72 h (control 0.91 ± 0.1 vs TNF-α-24 h 1.63 ± 0.09 vs TNF-α-72 h 0.63 ± 0.02, fold change, *p* < 0.0001; Fig. 3h and Supplementary Fig. 5a). Likewise, Gal-3 protein expression was also increased with TNF-α treatment at 24 h and restored by 72 h (control 1.00 ± 0.06 vs TNF-α-24 h 2.10 ± 0.15 vs TNF-α-72 h 0.98 ± 0.09, fold change, *p* < 0.0001, Fig. 3i and Supplementary Fig. 5b).

### Endothelial cells spontaneously form vascular networks within microfluidic chips that are impacted by the presence of trophoblasts and inflammatory conditions

**Fig. 4:**
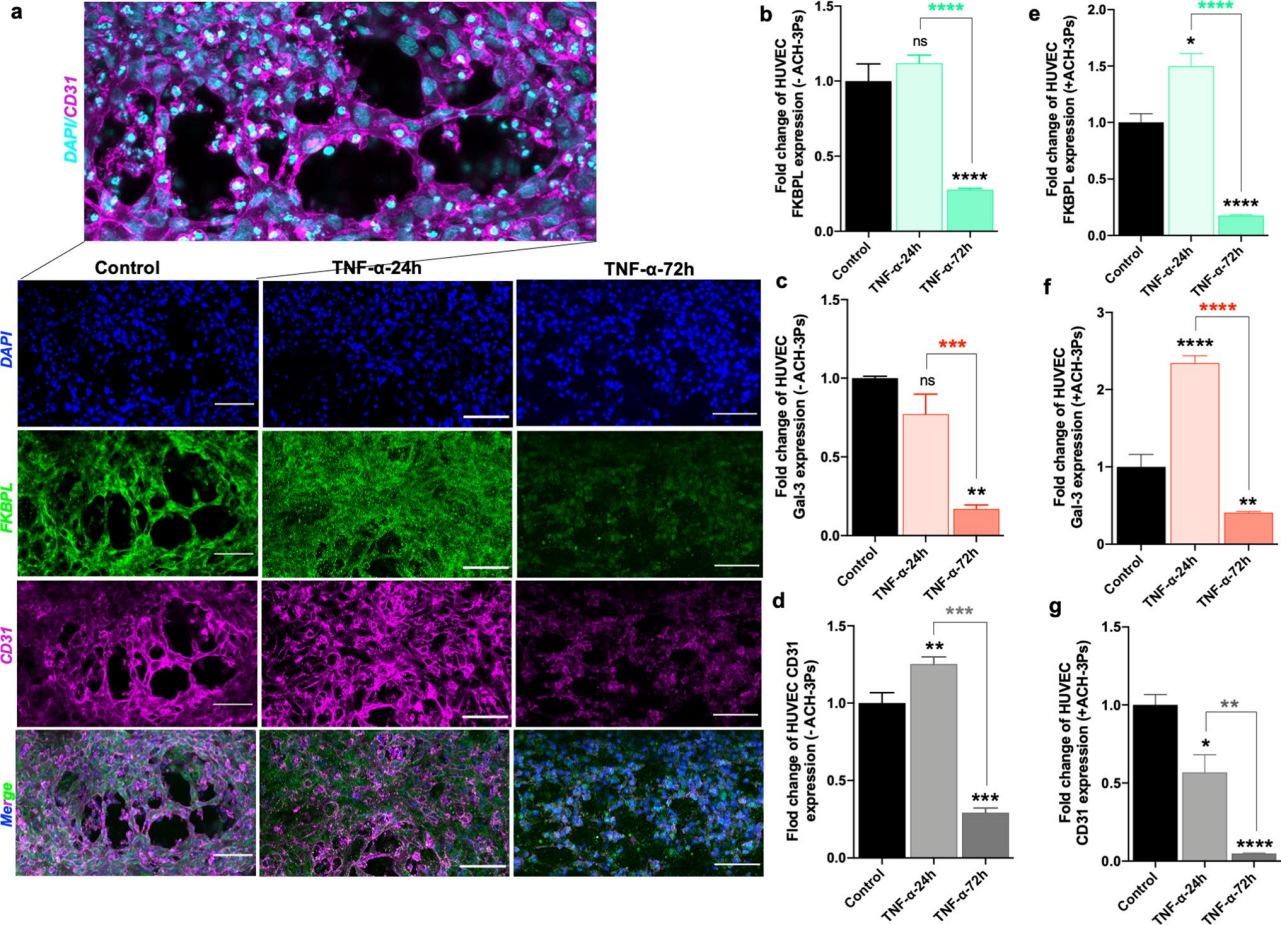
The presence of ACH-3Ps cells and inflammatory conditions impacts vasculare network formation and FKBPL, Gal-3 and CD31 expression in endothelial cells cultured in a microfluidic device. **a** In endothelial cells monoculture microfluidic setting, HUVECs were combined with collagen matrix (2.5 mg/mL) and added to the central channel of the microfluidic chips. In the co-culture set of chips, HUVECs were embedded within the central matrix channel and ACH-3Ps were added to the side channel. Chips were treated with TNF-α (10 ng/mL) for 24 or 72 h, with untreated cells as a control. Following 72 h of culture, chips were probed for immunofluorescent imaging of FKBPL, CD31 and Gal-3. **a** Representative images of cells stained for DAPI, FKBPL and CD31. **b** Fold change of FKBPL, **c** Gal-3 and **d** CD31 expression in HUVECs without ACH-3Ps. **e** Fold change of FKBPL, **f** Gal-3 and **g** CD31expression in HUVECs with ACH-3Ps. Data presented as mean ± SEM, scalebar represents 100 µm. Unpaired student’s *t* test and ordinary one-way ANOVA with Tukey post hoc test for normally distributed data and Mann–Whitney or Kruskal–Wallis post hoc test for non-normally distributed data; *n* = 3; *p < 0.05, **p < 0.01, ***p < 0.001, ****p < 0.0001

To investigate mechanisms of placental vascular dysfunction, our next step was to examine vascular network formation of HUVECs both in the presence and absence of ACH-3Ps, and/or inflammation. Confocal microscopy showed intricate vascular network formation by endothelial cells with junctions between branches (Fig. 4a). In the microfluidic environment containing just endothelial cells, 24-h TNF-α treatment had no effect on endothelial FKBPL protein expression; however, after 72 h, FKBPL protein expression was reduced by ~ 3.5-fold (control 1.00 ± 0.1 vs TNF-α-24 h 1.12 ± 0.05 vs TNF-α-72 h 0.28 ± 0.01, fold change, *p* < 0.0001; Fig. 4a, b). Similarly, there was no difference in endothelial Gal-3 protein expression within 24-h TNF-α treatment; however, after 72 h, Gal-3 protein expression was also reduced by ~ four-fold (control 1.00 ± 0.01 vs TNF-α-24 h 0.77 ± 0.13 vs TNF-α-72 h 0.17 ± 0.02, fold change, *p* = 0.0006; Fig. 4c and Supplementary Fig. 6b). Furthermore, there was a significant increase in CD31 protein expression of HUVECs following 24 h of TNF-α treatment, which was reduced significantly by 72 h (control 1.00 ± 0.07 vs TNF-α-24 h 1.25 ± 0.04 vs TNF-α-72 h 0.29 ± 0.02, fold change, *p* < 0.0001; Fig. 4a, d). However, in the co-culture system, the presence of trophoblast cells and TNF-α treatment increased the FKBPL expression of HUVECs following 24 h of TNF-α treatment which was reduced significantly by 72 h (control 1.00 ± 0.08 vs TNF-α-24 h 1.50 ± 0.11 vs TNF-α-72 h 0.18 ± 0.01, fold change, *p* < 0.0001; Fig. 4e and Supplementary Fig. 7a). Like FKBPL expression, TNF-α treatment for 24 h led to an initial increase in Gal-3 expression of HUVECs by ~ 2.5-fold that was significantly reduced by 72 h (control 1.00 ± 0.16 vs TNF-α-24 h 2.34 ± 0.09 vs TNF-α-72 h 0.41 ± 0.02, fold change, *p* < 0.0001; Fig. 4f and Supplementary Fig. 7b). In the presence of ACH-3Ps, a progressive decrease in CD31 expression following TNF-α treatment was observed (control 1.00 ± 0.07 vs TNF-α-24 h 0.57 ± 0.11 vs TNF-α-72 h 0.05 ± 0.004, fold change, *p* = 0.0004; Fig. 4g and Supplementary Fig. 7a) in the system.

**Fig. 5:**
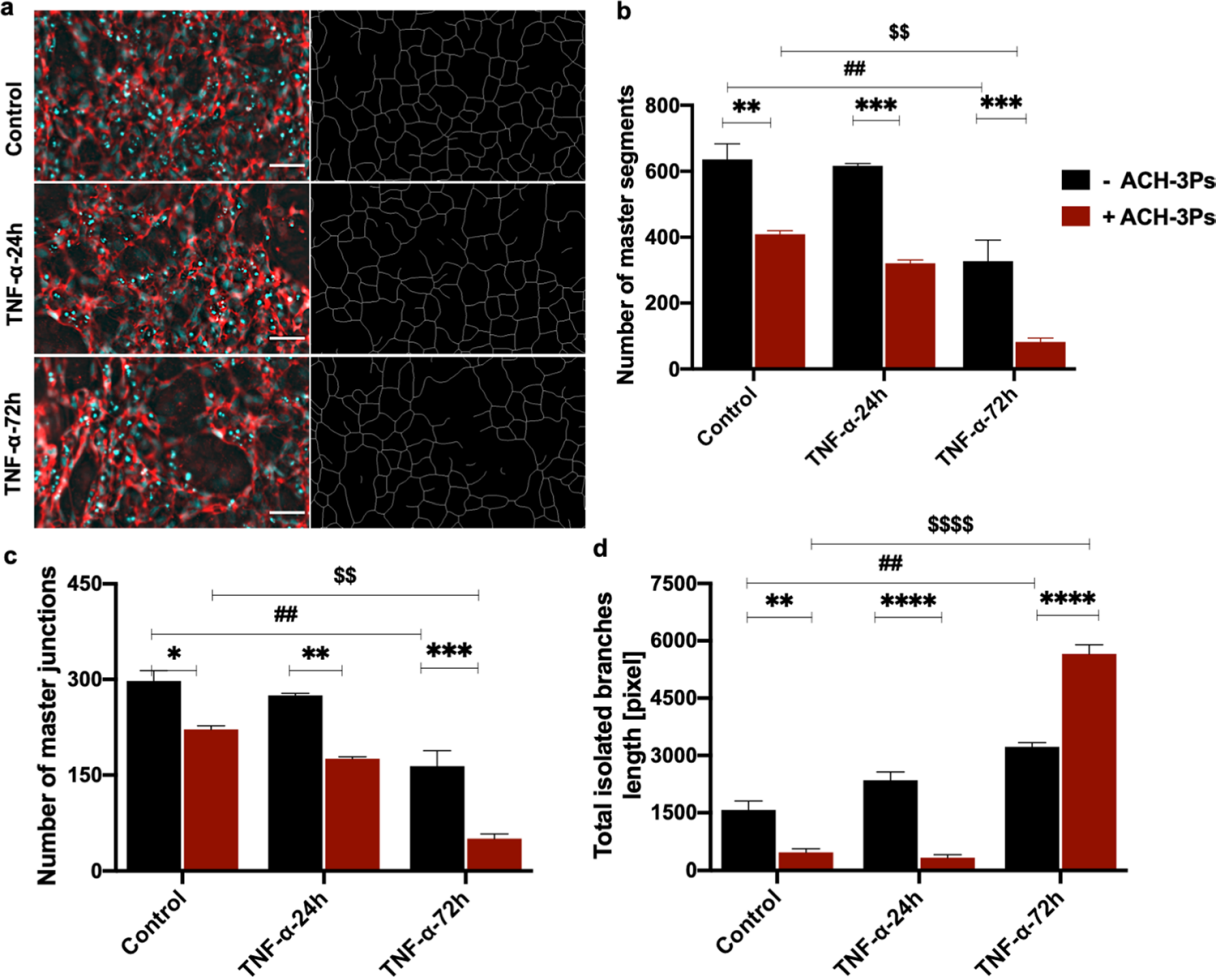
Quantification of vascular network formation. **a** Representative immunofluorescent images of HUVECs in microfluidic devices under different TNF-α conditions, that were analyzed using the Angiogenesis Analyzer ImageJ macro and their corresponding map outputs. Scalebar represents 100 µm. The **b** number of master segments, **c** number of master junctions and **d** total isolated branches length of HUVECs with and without ACH-3Ps in the system. Data presented as mean ± SEM. Ordinary two-way ANOVA with Tukey post hoc test; *n* = 3; **p < 0.01, ***p < 0.001, ****p < 0.0001

We also examined the 3D HUVECs monoculture and co-culture vascular network structures within the microfluidic environment by measuring the number of master segments, master junctions and total isolated branches using an Angiogenesis Analyzer macro [54]. There was a significant difference in HUVEC monoculture and co-culture vascular network structures and following 72 h TNF-α treatment including the number of master segments (-ACH3-Ps vs + ACH-3Ps; Control: 636.3 ± 47.32 vs 409.8 ± 10.7, TNF-α-24 h: 616.7 ± 6.7 vs 320.6 ± 10.4, TNF-α-72 h: 327.7 ± 63.7 vs 82.3 ± 11.68, *p* < 0.0001; Fig. 5a, b), number of master junctions (-ACH-3Ps vs + ACH-3Ps; Control: 297.3 ± 16.5 vs 221.3 ± 5.8, TNF-α-24 h: 274.7 ± 3.3 vs 175.3 ± 3.3, TNF-α-72 h: 163.7 ± 24.6 vs 50.33 ± 7.6, *p* < 0.0001; Fig. 5c) and total isolated branches (-ACH-3Ps vs + ACH-3Ps; Control: 1575 ± 232.4 vs 468.3 ± 93.8, TNF-α-24 h: 2352 ± 219.3 vs 330 ± 76.6, TNF-α-72 h: 3223.7 ± 114.6 vs  5656.3± 240.6, *p* < 0.0001; Fig. 5d). Most of these results show reduction in vascular network formation in our placenta-on-a-chip model in the presence of trophoblast cells or TNF-α (Fig. 5b, c), which is reflective of SUA remodeling during placental development. However, prolonged TNF-α treatment (72 h) reduced and impaired organized vascular network formation, measured by the increase in isolated branches, which is even more pronounced in the presence of ACH-3Ps (Fig. 5d).

## Discussion

Despite extensive research, there has been a lack of definitive prophylactic and curative treatment options for preeclampsia during pregnancy. This has, in part, been due to difficulties in obtaining human samples of the early placenta and the lack of biologically relevant model systems of this human disease. Consequently, establishing a reliable and representative model of the early placenta to study mechanisms leading to preeclampsia remains important toward developing better monitoring and treatment strategies for women and babies affected by preeclampsia. In this study we utilized a placenta-on-a-chip model that recapitulates aspects of placental development in inflammatory conditions including ACH-3P trophoblast migration and invasion, and endothelial vascular network development. Importantly, in our study we showed that (i) there is an upregulation of novel angiogenesis- and inflammation-related proteins, FKBPL and Gal-3, in both placenta and plasma samples collected from women with preeclampsia compared to normotensive controls, (ii) endothelial and trophoblast interactions can affect changes in FKBPL and Gal-3 protein expression patterns and (iii) inflammation and upregulation of FKBPL and Gal-3 is associated with impaired vascular network formation. All these aberrant placental changes can contribute to preeclampsia.

As introduced earlier, FKBPL has been shown to have roles in the regulation of steroid receptor signaling, cell differentiation and inhibition of angiogenesis [25–31]. In our previous study, we observed that plasma FKBPL was reduced early in pregnancy (15-week gestation) in women who proceeded to develop preeclampsia, while following diagnosis FKBPL was significantly increased in plasma and placentae compared to healthy controls [22]. Here, we confirmed that following diagnosis of preeclampsia, FKBPL expression was significantly increased in the plasma and placentae of our new validation group of women with preeclampsia compared to normotensive controls. In addition, we have recently shown the increased expression of FKBPL in the hearts of pregnant rats with reduced uterine perfusion pressure (RUPP), an in vivo model of preeclampsia, and cardiac spheroids treated with plasma from women with preeclampsia [34]. Given that FKBPL has anti-angiogenic properties, increased levels of FKBPL are associated with restricted angiogenesis, which is a hallmark of preeclampsia.

Furthermore, in our study we observed significantly increased levels of Gal-3 in placenta samples from women with preeclampsia compared to controls. Gal-3 is a known immunomodulatory protein and its specific structure facilitates the binding of ECM glycans to cell surfaces during tissue remodeling [36, 66]. On one hand, this altered phenotype may be reflective of adverse placental tissue remodeling leading to fibrosis, induced by increased inflammation, which are characteristic features of preeclampsia [67], although this would require further tissue analysis. On the other hand, Gal-3 has also been shown to promote invasion and differentiation of trophoblasts, with EVTs from preeclamptic placentae expressing higher levels of Gal-3; however, evidence of lineage-specific effects are conflicting [38–40, 68–71]. In addition, we found circulating concentration of Gal-3 in the plasma of women with preeclampsia to be significantly increased, in line with published literature [48–50]. Gal-3 has been shown to not only regulate tissue remodeling particularly in cardiovascular disease, but an increase in plasma Gal-3 correlates with conventional cardiovascular risk factors [72, 73]. Therefore, the increased levels of Gal-3 in the plasma from women with preeclampsia could represent an important mechanism leading to maternal cardiovascular remodeling and increased risk of future cardiovascular disease. While in our study there was no statistically significant correlation between Gal-3 secretion and preeclampsia, this could have been influenced by gestational age and suggests that Gal-3 expression changes throughout the time course of pregnancy, which has been previously observed [74, 75].

In our 2D monoculture experiments, we observed that inflammatory cytokine TNF-α upregulated both FKBPL and Gal-3 in trophoblasts and endothelial cells individually. To our knowledge, this regulatory effect of TNF-α on the expression of FKBPL has not previously been shown. Along with emerging evidence identifying FKBPL’s role in regulating nuclear factor-κB (NF-κB) signaling, this could contribute to our understanding of the importance of FKBPL in inflammatory pathways [32]. In addition, various studies suggest that Gal-3 is under the positive control of TNF-α [76–78]. Within the context of pregnancy, in in vitro trophoblast experiments, TNF-α and Gal-3 have been shown to regulate each other, likely by a positive feedback mechanism, and may contribute to inflammatory processes in preeclampsia [79].

Several 3D-microfluidic models of the placenta have been developed [80]. For example, Lee et al*.* generated a placenta-on-a-chip model by adding human choriocarcinoma cell line, JEG-3, and HUVECs in media channels separated by an ECM channel [81]. However, the purpose of this model was not to investigate the migration and interaction of these cell types, but rather their ability to form a permeable barrier. On the other hand, Abbas et al*.* developed a microfluidic model to study the migratory characteristics of EVTs by embedding primary human first trimester trophoblasts in a central ECM channel [82]. These cells were exposed to a granulocyte–macrophage colony-stimulating factor gradient collected from activated decidual natural killer cells, which stimulated increased and more directional invasion toward the secreted factors. Considering this, we aimed to study the direct invasion and interaction of trophoblast-like ACH-3P cells and human endothelial cells to elucidate novel mechanisms of early placental development in inflammatory conditions [83]. We selected ACH-3P cells due to their ability to spontaneously differentiate into syncytiotrophoblast-like cells capable of secreting human chorionic gonadotropin and into an HLA-G + EVT-reflective population, hence closely resembling primary first trimester trophoblasts [58]. Syncytiotrophoblasts are believed to have critical role in both normal and dysfunctional pregnancy through the secretion of extracellular vesicles that regulate the maternal immune system during the pregnancy [84, 85]. These extracellular vesicles are increased in preeclampsia and likely contribute to the endothelial dysfunction characteristic of the disease [84, 85]. In addition, EVTs were shown to play an active role in remodeling the SUAs by inducing endothelial apoptosis through Fas/FasL interaction [86]. In the context of trophoblast cells, we have shown that TNF-α treatment induced an initial reduction and then an increase in ACH-3Ps’ FKBPL and Gal-3 expression in the absence of HUVECs. In contrast, the presence of endothelial cells adversely affects the ACH-3Ps, indicating a potential regulatory role of these cells on trophoblast FKBPL and Gal-3 expression which in turn enhance the trophoblast migration into the endothelial networks. A recent study has shown that TNF-α treatment for 24 h not only increases the oxidative stress and apoptosis, but also promotes cell migration and invasion in primary trophoblast cells through NF-κB activation [83].

When observing the response of endothelial cells, the initial increase in FKBPL protein expression by HUVECs correlated with their diminished angiogenic potential, as demonstrated by reduced master segments and master junctions, reflecting their inability to form new branches from existing structures. This effect is consistent with previous findings that *Fkbpl* knockout mice are embryonic lethal, while *Fkbpl* heterozygous knockdown mouse embryos exhibit pro-angiogenic phenotype with impaired/leaky vasculature, strongly suggesting that FKBPL plays an essential role in developmental and physiological angiogenesis [28]. Whether the subsequent reduction in FKBPL expression at 72 h indicates a compensatory decrease to enable the stimulation of angiogenesis requires further investigation. Gal-3 expression of HUVECs exhibited a similar expression pattern to FKBPL with an initial increase when trophoblasts were present, followed by a significant reduction by 72 h. This initial increase could also be due to a compensatory effect to stimulate angiogenesis. In the context of cancer, Gal-3 has been shown to promote angiogenesis and enable metastasis [87, 88].

In our placenta-on-a-chip model, we demonstrated the formation of stable vascular endothelial networks similar to previously published studies [89–92]. We further demonstrated that TNF-α treatment in HUVECs monocultures initially increased the expression of endothelial marker CD31, whereas CD31 expression was overall decreased in endothelial and trophoblast co-culture conditions. Pan-endothelial CD31 is an important vascular cell adhesion and signaling molecule that regulates endothelial cell migration, survival and maintenance of the endothelial cell permeability barrier [93]. Whether the reduction we noted is due to endothelial cell death induced by TNF-α [94, 95], replacement by trophoblast cells [86, 96], or simply reduced expression in functional endothelial cells remains to be answered. However, placentae and decidua from women with preeclampsia, particularly with decidual vasculopathy, have been shown to express reduced levels of CD31, an indication of failed angiogenesis [97]. In line with this, the addition of TNF-α to our microfluidic system increased FKBPL and Gal-3 expression and impaired the ability of HUVECs to form continuous vascular networks, as seen in the increase in the length of isolated branches, which are unable to establish appropriate junctions with nearby branches. According to our data, an initial upregulation of antiangiogenic-related protein, FKBPL, can interfere with the inflammation-induced angiogenesis process which leads to vascular complications in the preeclamptic placenta.

Our findings demonstrate that this 3D microfluidic model is an adaptable system for investigating complex pathophysiology under different treatments and conditions, as it provides a highly controllable, dynamic microenvironment and permits the observation of cellular interactions and behavior in real-time. One limitation of the model we present is the potential influence of the choriocarcinoma component of the ACH-3P cell line, and the absence of primary cells and human uterine microvascular endothelial cells of the SUA. However, the incorporation of the immortalized ACH-3P line enabled us to generate a low-risk, low-cost and reproducible model of first trimester trophoblast-like and endothelial cell interactions in early placentation. To improve the physiological or pathological relevance, this 3D microfluidic model should be enhanced to include human tissue-derived ECM, primary first trimester trophoblasts, human uterine microvascular endothelial cells, stromal, glandular epithelial and immune cells or the addition of other key factors in preeclampsia, such as hypoxia or sFlt-1 overexpression to reach its full potential.

## Conclusions

We developed a proof-of-concept placenta-on-a-chip model incorporating immortalized first trimester trophoblasts and endothelial cells, which are key cells for placental development and growth. We developed this model to resemble a key feature of the preeclamptic placenta by including an important inflammatory factor, TNF-α, secreted by immune cells and shown to be increased in preeclampsia. This system can be made more complex by the incorporation of other cell types involved in placentation including fibroblasts, pericytes, decidual natural killer cells and macrophages to broaden our understanding of the pathogenesis of preeclampsia. We also validated this model by evaluating the emerging FKBPL and Gal-3 inflammatory and anti-angiogenic mechanisms in placental development and growth, compared to their secretion and expression in plasma and placental samples from women with preeclampsia. The application of microfluidic systems in evaluating angiogenesis and trophoblast invasion, hallmark features of successful pregnancy, was also demonstrated. Our placenta-on-a-chip model could be utilized in the future for biomarker discovery. Importantly, this platform will allow high-throughput screening of various therapeutic agents for conditions such as preeclampsia that currently have no definitive treatments. Moreover, this platform can reduce the use of animals in research investigating placental development and enable safe, low-cost, and reliable testing of novel therapeutic agents for pregnancy conditions affected by aberrant placentation.

## Supplementary Information

Below is the link to the electronic supplementary material.

Supplementary file1 (DOCX 16484 KB)

## Data Availability

All the data generated or analysed during this study are available from the corresponding author upon resealable request.
