# Supplementary material for: A placenta-on-a-chip model to determine the regulation of FKBPL and galectin-3 in preeclampsia
Source: Cell Mol Life Sci. 2023 Jan 18;80(2):44. doi: 10.1007/s00018-022-04648-w (PMC9849194; doi:10.1007/s00018-022-04648-w)
Supplement: Supplementary file 1 — Supplementary file1 (DOCX 16484 KB) [file 18_2022_4648_MOESM1_ESM.docx]

**Supplementary Information**

A placenta-on-a-chip model to determine the regulation of FKBPL and galectin-3 in preeclampsia

*Sahar Masoumeh Ghorbanpour^1,2#^, Claire Richards^1,2#^, Dillan Pienaar^1^, Kimberly Sesperez^1^, Hamidreza Aboulkheyr Es.^3^, Valentina Nikolic^4^, Natasa Karadzov Orlic^5,6^, Zeljko Mikovic^5,6^, Milan Stefanovic^4,7^, Zoran Cakic^8^, Abdelrahim Alqudah^9^, Louise Cole^10^, Catherine Gorrie^1^, Kristine McGrath^1^, Mary M. Kavurma^11,12^, Majid Ebrahimi Warkiani^2,3^, Lana McClements*^1,2^*

1. School of Life Sciences, Faculty of Science, University of Technology Sydney, NSW, Australia

2. Institute for Biomedical Materials and Devices, Faculty of Science, University of Technology Sydney, NSW, Australia

3. School of Biomedical Engineering, Faculty of Engineering and Information Technology, University of Technology Sydney, NSW, Australia

4. Department of Pharmacology and Toxicology & Department of Internal Medicine - Gynaecology, Medical Faculty, University of Nis, Nis, Serbia

5. Department of Gynaecology and Obstetrics, Narodni Front, Belgrade, Serbia

6. Medical Faculty, University of Belgrade, Belgrade, Serbia

7. Department of Gynaecology and Obstetrics, Clinical Centre Nis, Nis, Serbia

8. Department of Gynaecology and Obstetrics, General Hospital of Leskovac, Leskovac, Serbia

9. Department of Clinical Pharmacy and Pharmacy Practice, Faculty of Pharmaceutical Sciences, The Hashemite University, Zarqa, Jordan

10. The iThree Institute, Faculty of Science, University of Technology Sydney, NSW, Australia

11. Heart Research Institute, Newtown, Sydney, NSW, Australia

12. The University of Sydney, Sydney, NSW, Australia

E-mail: [lana.mcclements@uts.edu.au](mailto:lana.mcclements@uts.edu.au)

# Authors contributed equally

* Corresponding author

**Keywords:** microfluidics, placental development, preeclampsia, vascular remodeling, FKBPL, galectin-3

**Supplementary Table 1.** Clinical characteristics of normotensive pregnancies and pregnancies with established preeclampsia whose placentae were analyzed

|  | **Control (n= 17)** | **Preeclampsia (n=30)** | **P value** |
| --- | --- | --- | --- |
| Age (years) | 31.83±4.16 | 36.64±3.69 | **0.0269** |
| Gestational age at delivery (weeks) | 39.42±0.92 | 32.87±3.48 | **0.0002** |
| BMI | 25.65±4.05 | 30.61±5.13 | 0.0598 |
| sBP (mmHg) | 108±11.69 | 154.5±31.66 | **0.0004** |
| dBP (mmHg) | 68.33±9.83 | 96.54±11.79 | **0.0004** |
| MABP (mmHg) | 81.50±9.91 | 114.4±17.44 | **0.0005** |
| Gravidity | 1.5 (1-3) | 2.36 (1-3) | 0.0613 |

*All clinical characteristics are given as mean ± SD.* *Bold indicates statistical significance (p<0.05)*.

*Key: BMI, body mass index; sBP, systolic blood pressure; dBP, diastolic blood pressure; MABP, mean arterial blood pressure.*

**Supplementary Table 2.** Clinical characteristics of normotensive pregnancies and pregnancies with established preeclampsia whose plasma was collected

|  | **Control (n=6)** | **Preeclampsia (n=11)** | **P value** |
| --- | --- | --- | --- |
| Age (years) | 31.24 ±3.93 | 33.31±5.89 | 0.2036 |
| Gestational age at delivery (weeks) | 39.02±1.59 | 33.47±3.47 | **<0.0001** |
| BMI | 24.06±5.38 | 27.59±4.39 | **0.0149** |
| sBP (mmHg) | 113.5±8.618 | 151.5±24.15 | **<0.0001** |
| dBP (mmHg) | 72.35±8.31 | 99.12±12.13 | **<0.0001** |
| MABP (mmHg) | 85.47±8.08 | 116.5±15 | **<0.0001** |
| Gravidity | 1.76 (1-3) | 1.86 (1-5) | 0.9702 |

*All clinical characteristics are given as mean ± SD.* *Bold indicates statistical significance (p<0.05)*.

*Key: BMI, body mass index; sBP, systolic blood pressure; dBP, diastolic blood pressure; MABP, mean arterial blood pressure.*

**
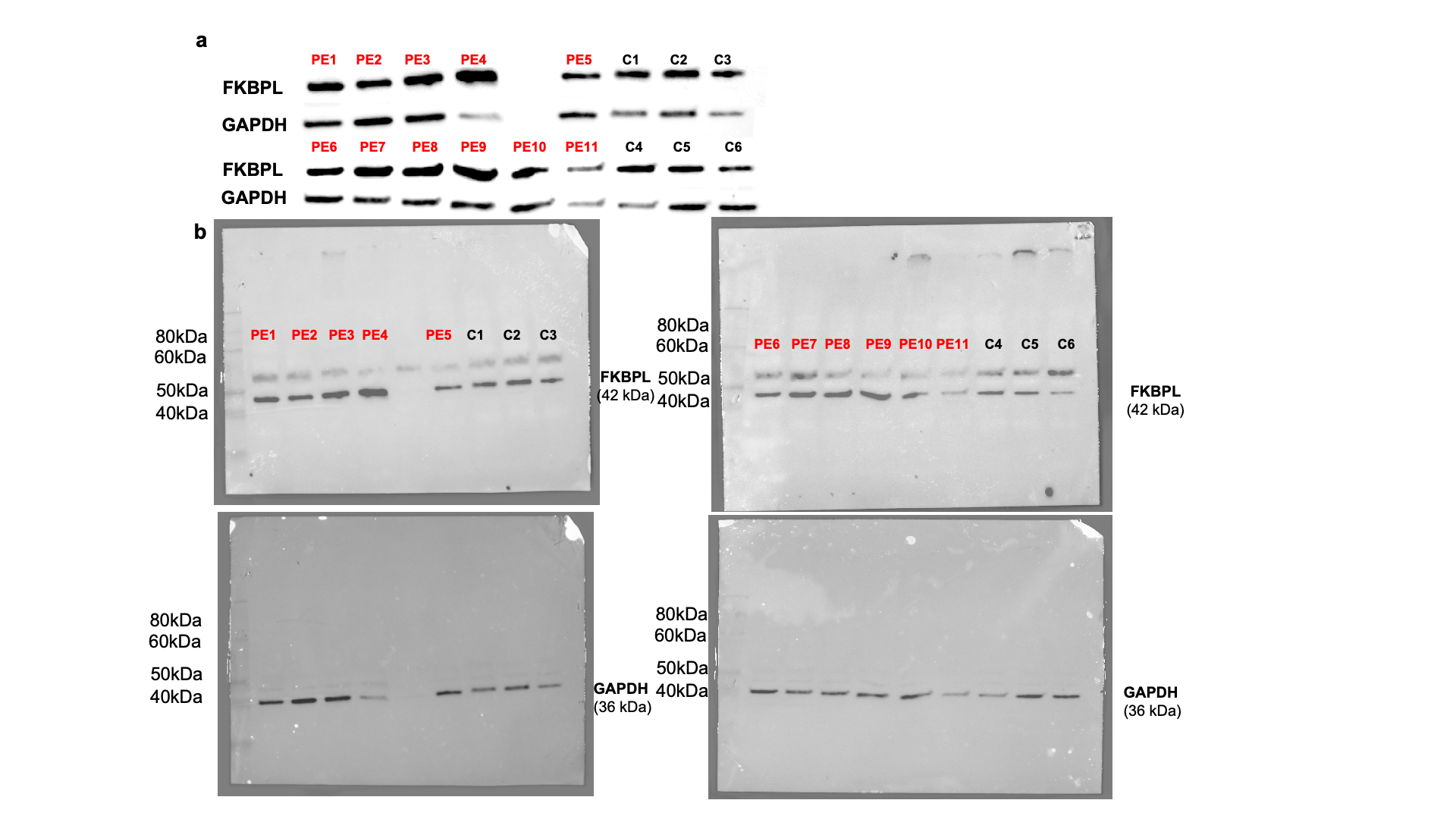
**

**Supplementary Fig. 1 FKBPL is increased in the placentae of women with preeclampsia.** Protein lysates were generated from placental tissue collected from women with preeclampsia or normotensive controls. (**a**) FKBPL expression was determined by Western Blotting and normalized to GAPDH, the loading control. (**b**) Raw chemiluminescent Western Blot image. PE, preeclampsia; C, Control (healthy patient).


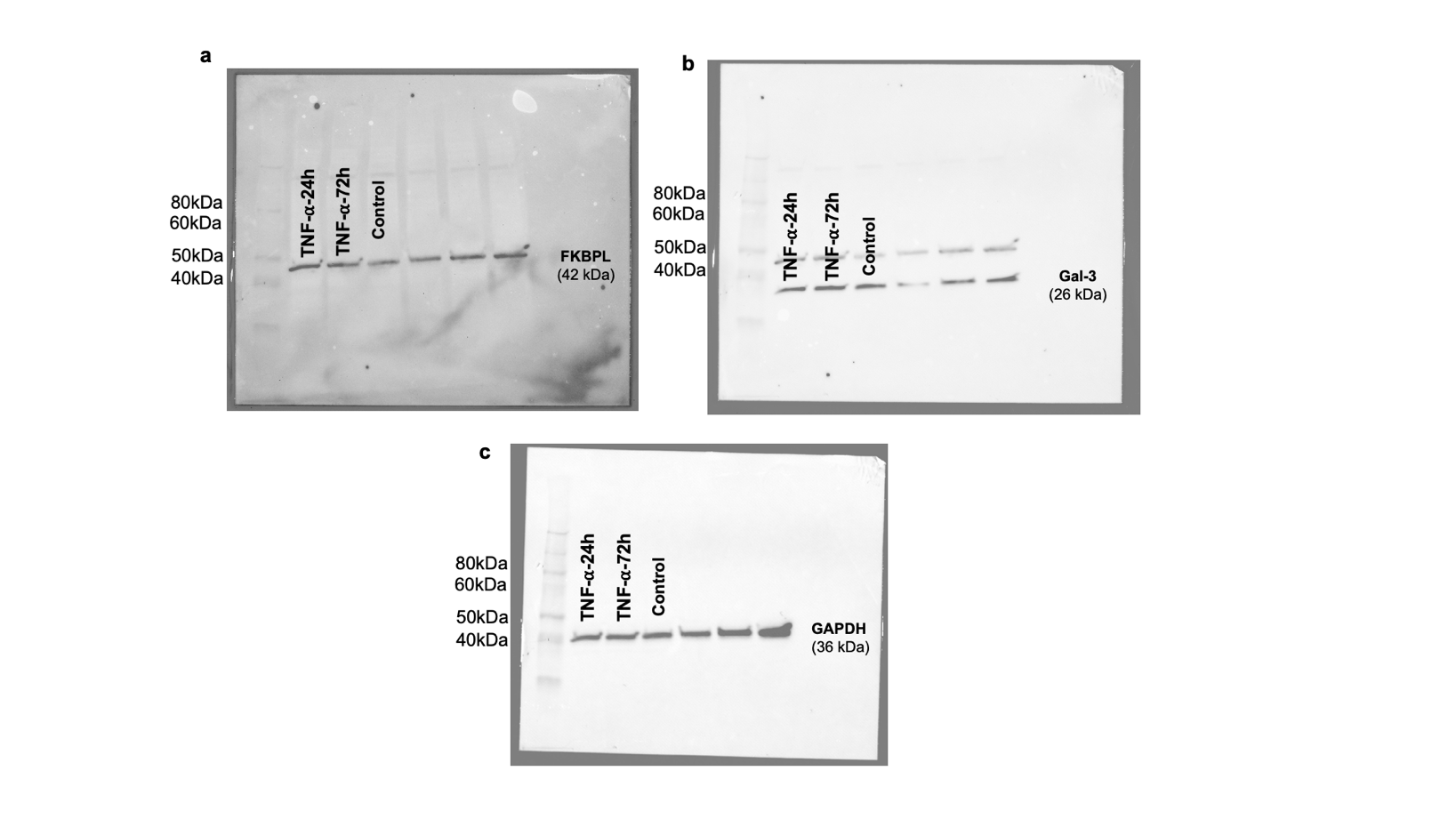


**Supplementary Fig. 2** **TNF-α treatment of ACH-3Ps in 2D monocultures alters FKBPL and Gal-3**. (**a-c**) The full blots of ACH-3Ps protein lysate expression of FKBPL and Gal-3. ACH-3Ps exposed to tumor necrosis factor alpha (TNF-α, 10ng/ml) for 24 or 72 hours. Control, untreated. GAPDH, loading control.

**
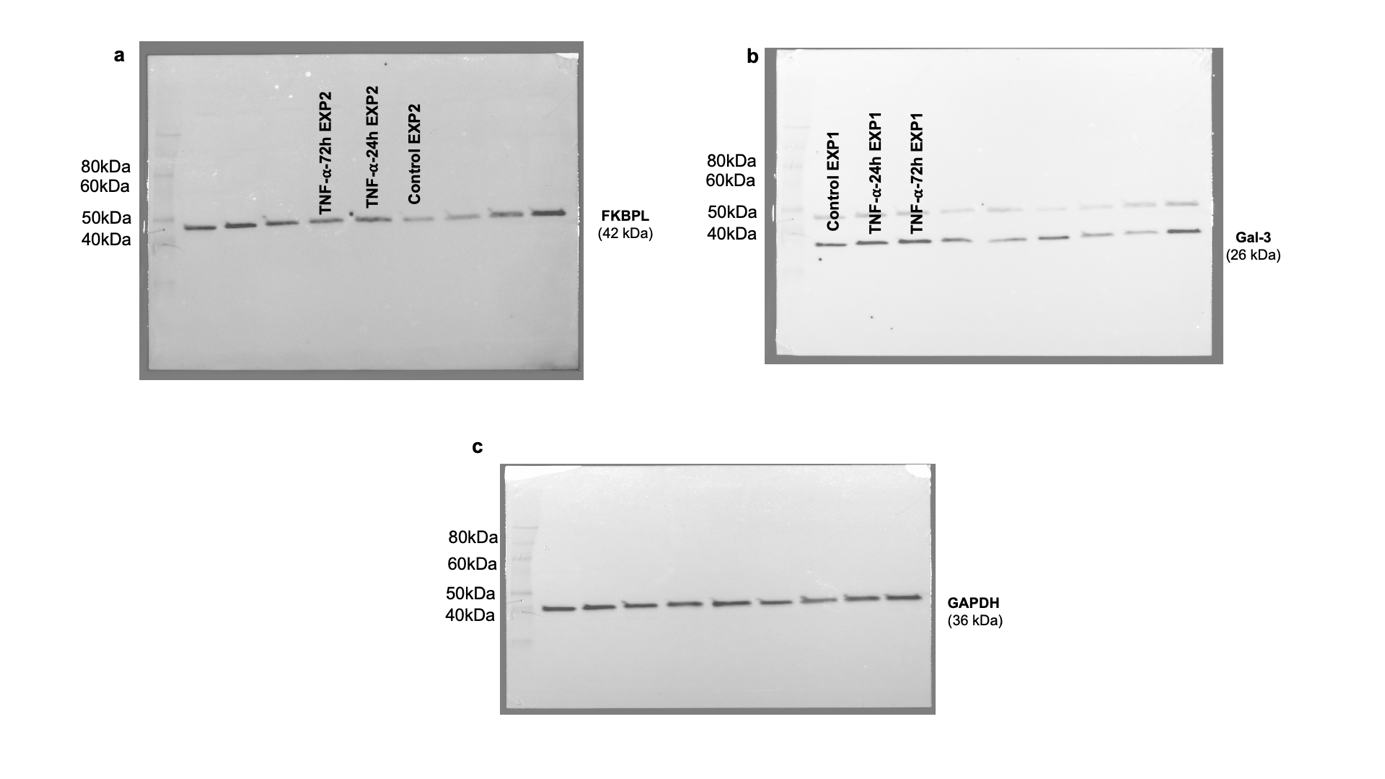
**

**Supplementary Fig. 3** **TNF-α treatment of HUVECs in 2D monocultures alters FKBPL and Gal-3**. (**a-c**) The full blots of HUVECs protein lysate expression of FKBPL and Gal-3. ACH-3Ps exposed to tumor necrosis factor alpha (TNF-α, 10ng/ml) for 24 or 72 hours. Control, untreated. GAPDH, loading control.

**
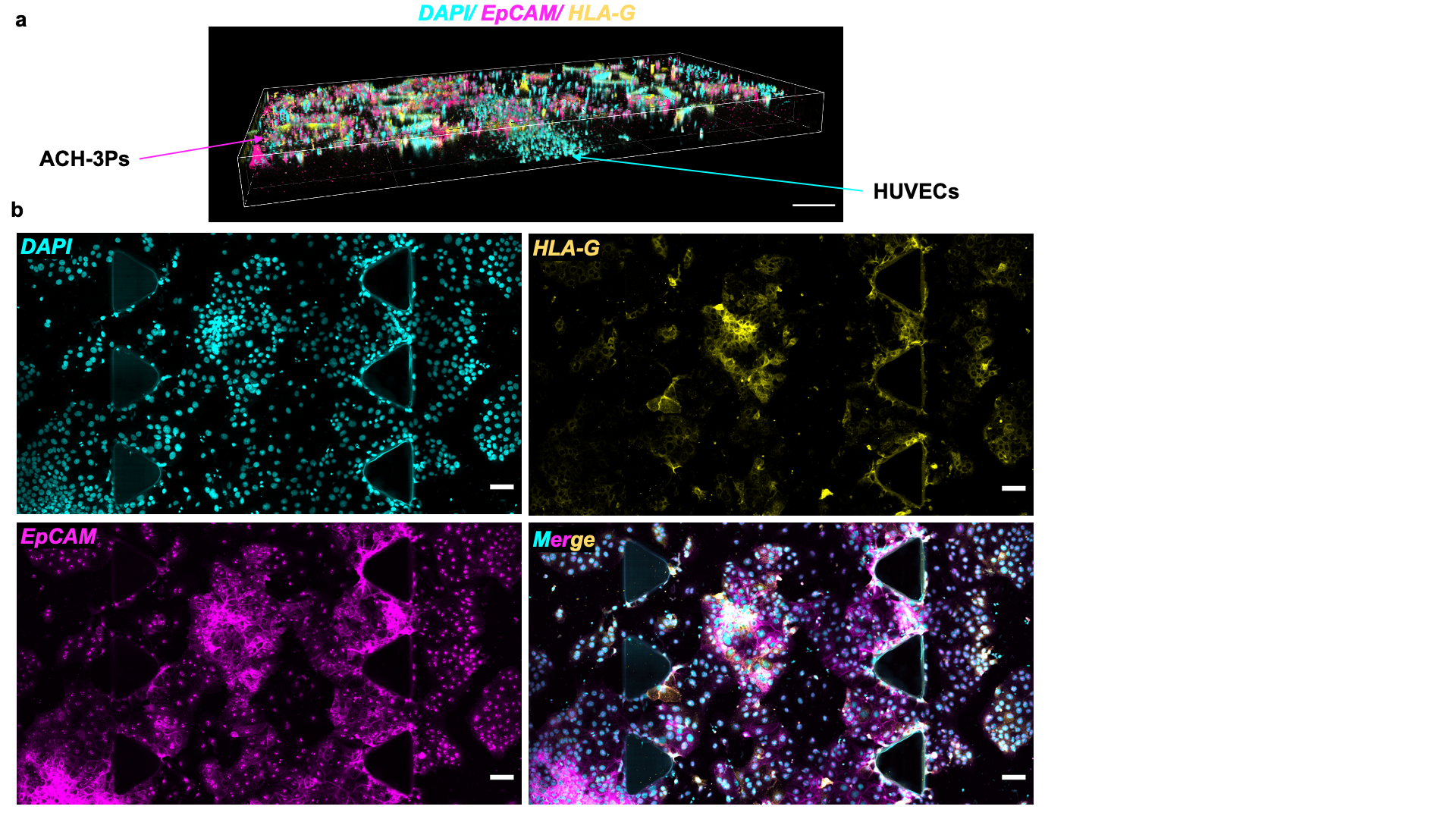
Supplementary Fig. 4 First trimester stem-like trophoblast cells (ACH-3Ps) can differentiate from villous cytotrophoblasts into extravillous trophoblasts in co-culture microfluidic chip.** In the co-culture set of chips, HUVECs were embedded within the center matrix channel and ACH-3Ps were added to the side channel. (**a**) Representative immunofluorescent volume view images of trophoblast cells and endothelial cells in co-culture microfluidic chip. (**b**) ACH-3P labelled for EpCAM (magenta; villous cytotrophoblasts), HLA-G (yellow; extravillous trophoblasts) and DAPI (cyan). Scalebars represent 100µm.

**
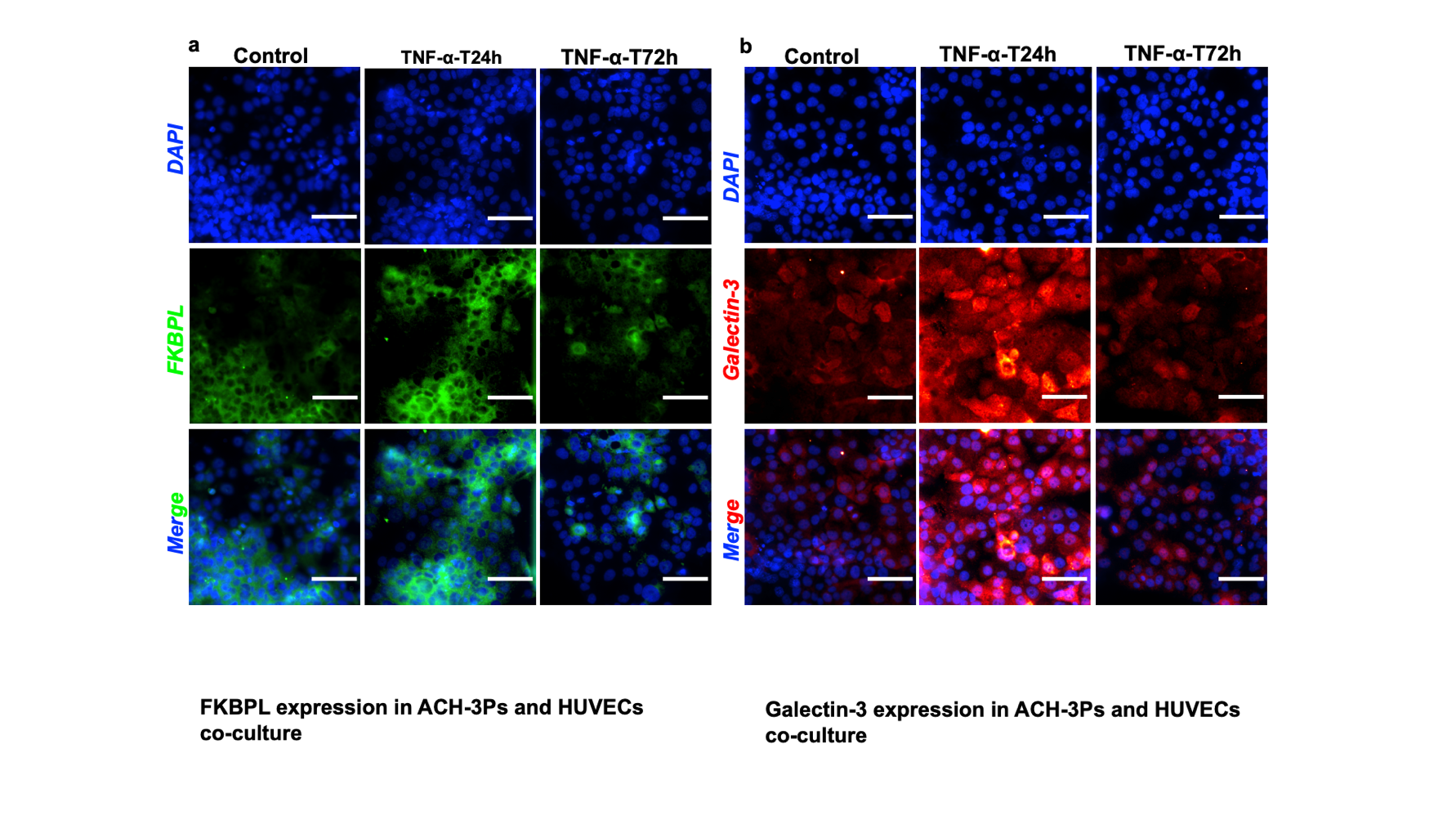
**

**Supplementary Fig. 5** **HUVECs presence and TNF-α modify ACH-3P FKBPL and Gal-3 expression in a endothelial and trophoblast co-culture microfluidic chip.** In the co-culture set of chips, HUVECs were embedded within the center matrix channel and ACH-3Ps were added to the side channel. Chips were treated with TNF-α (10ng/ml) for 24 or 72 hours, with untreated cells as a control. Following 72 hours of culture, chips were probed for immunofluorescent imaging of FKBPL and Gal-3. (**a**) And (**b**) Representative immunofluorescence (IF) images of ACH-3Ps for quantification of FKBPL and Gal-3 expression, respectively. Scalebars represent 100µm.

**
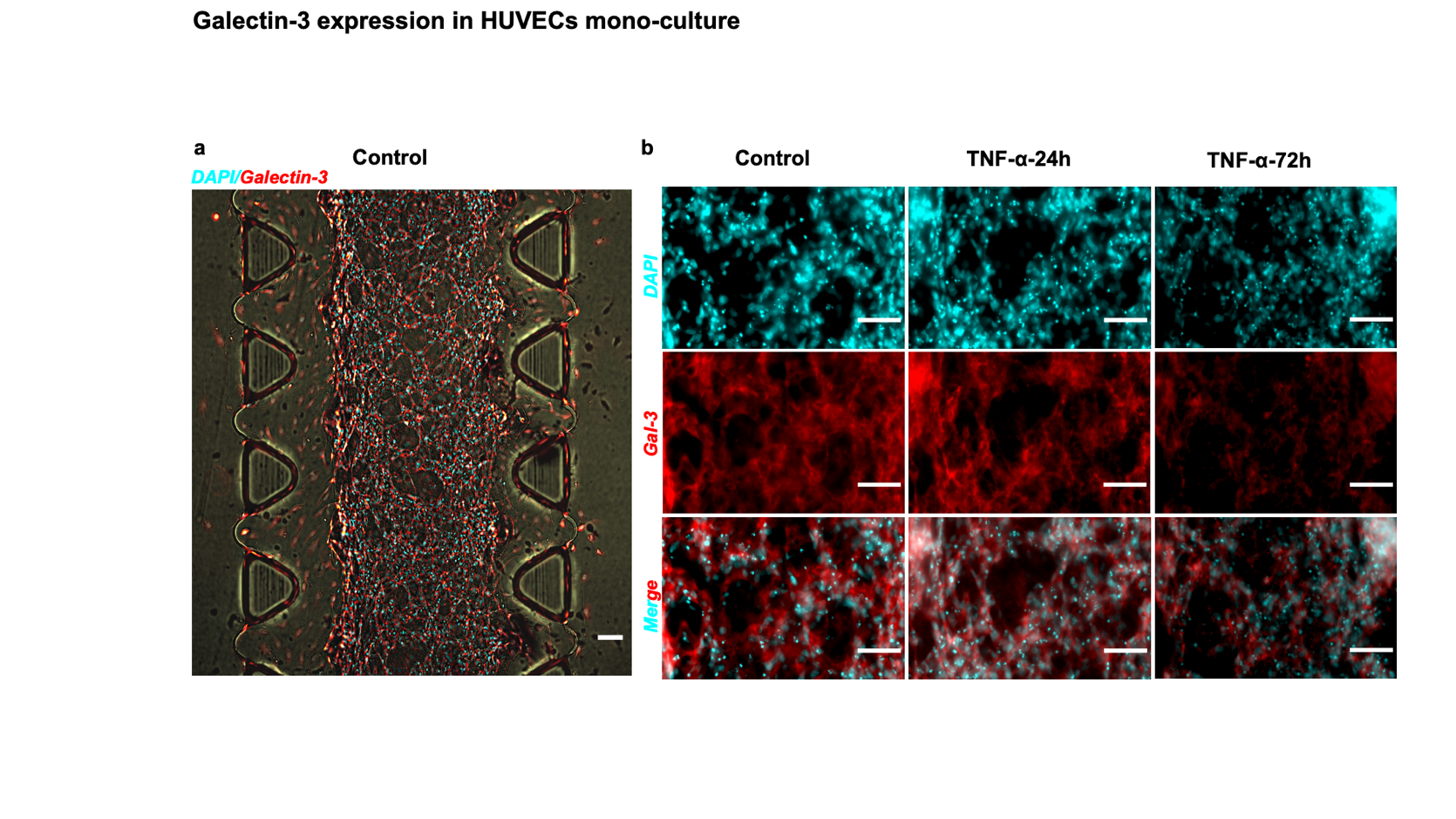
**

**Supplementary Fig. 6** **Inflammatory conditions impact Gal-3 expression in endothelial cells monoculture in a microfluidic device**. HUVECs were IF stained for Galectin-3 and Dapi. Chips were treated with TNF-α (10ng/ml) for 24 or 72 hours, with untreated cells as a control. (a) HUVECs were combined with collagen matrix (2.5mg/ml) and added to the central channel of microfluidic chips. Representative immunofluorescent images of HUVECs vascular network formation in microfluidic devices. (b) Representative immunofluorescence (IF) images of HUVECs for quantification of Gal-3 expression, respectively. Scalebars represent 100µm.


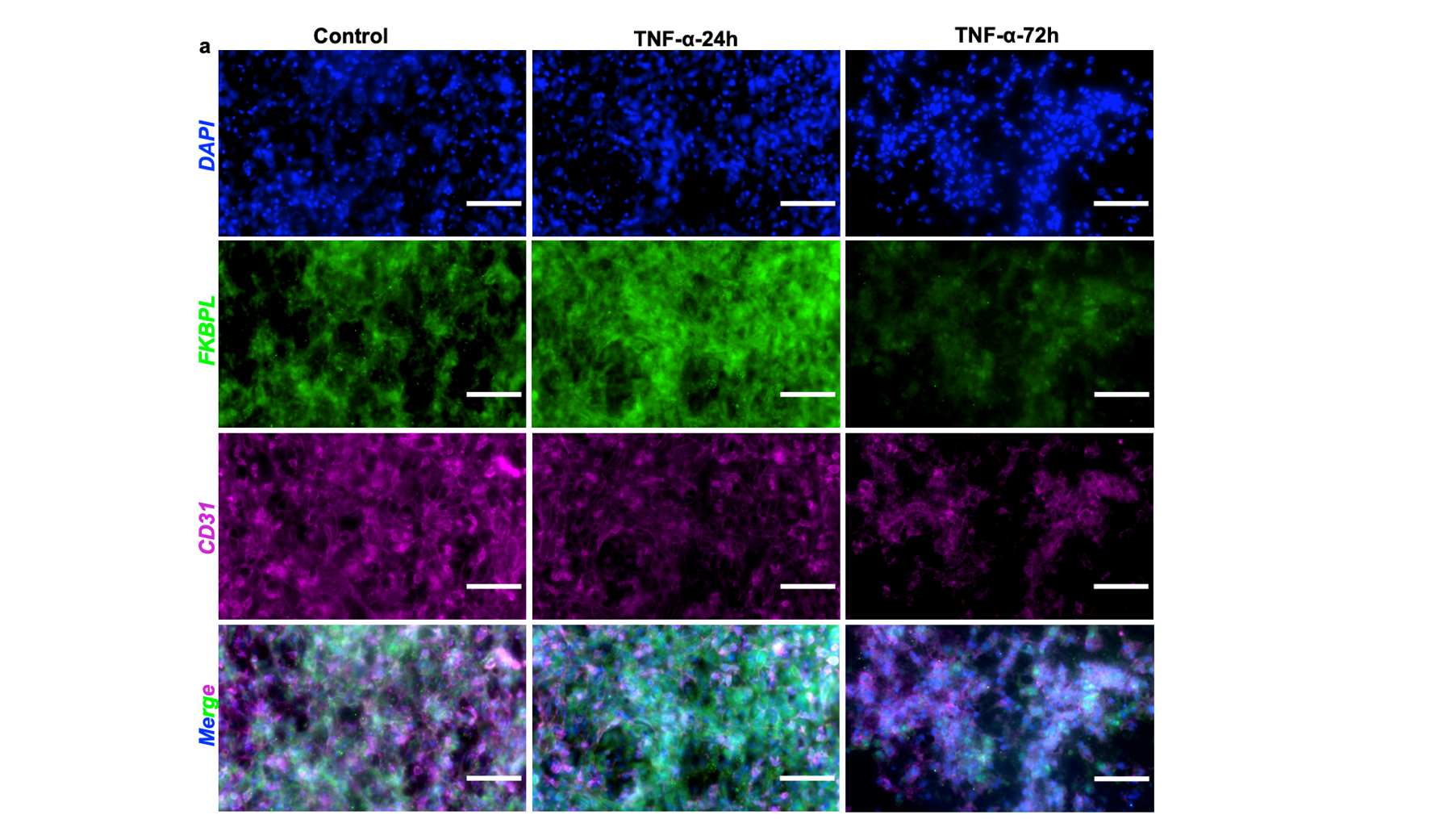


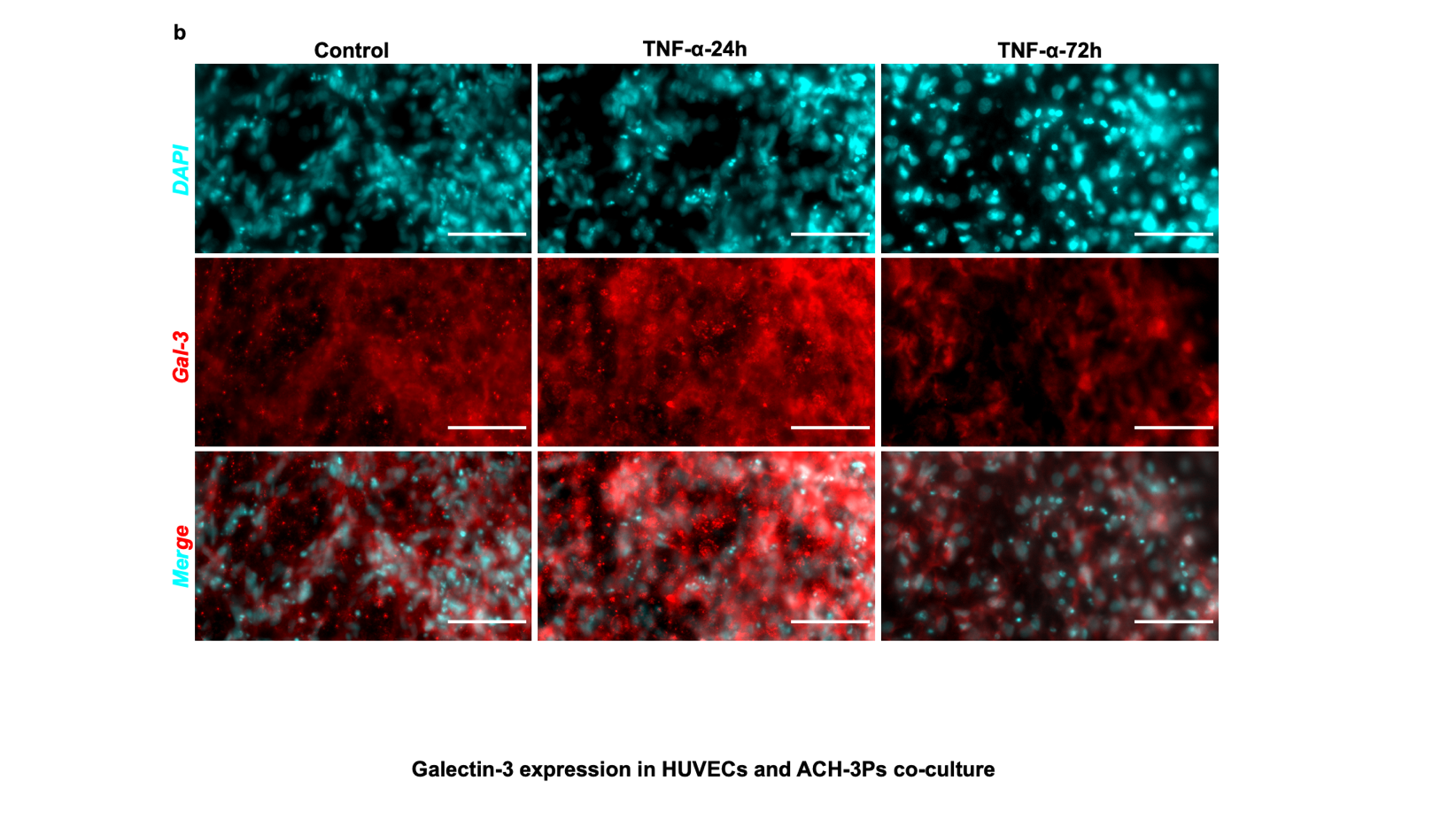


**Supplementary Fig. 7** **The presence of ACH-3Ps cells and inflammatory conditions impact FKBPL, CD31 and Galectin-3 expression in endothelial cells co-cultured in a microfluidic device.** (a) and (b) In the co-culture set of chips, HUVECs were combined with collagen matrix (2.5mg/ml) and added to the central channel of microfluidic chips and ACH-3Ps were added to the side channel. Chips were treated with TNF-α (10ng/ml) for 24 or 72 hours, with untreated cells as a control. Following 72 hours of culture, chips were probed for immunofluorescent imaging of FKBPL, CD31, Gal-3 and Dapi. (a) Representative immunofluorescence (IF) images of HUVECs for quantification of FKBPL and CD31. (b) Representative immunofluorescence (IF) images of HUVECs for quantification of Galectin-3. Scalebars represent 100µm.
